# Topological Properties of Co-Occurrence Networks in Published Gene Expression Signatures

**DOI:** 10.4137/bbi.s518

**Published:** 2008-04-17

**Authors:** Heiko Muller, Francesco Acquati

**Affiliations:** 1 The FIRC Institute of Molecular Oncology Foundation, Via Adamello 16, 20139 Milan, Italy; 2 Department of Experimental Oncology, European Institute of Oncology, Via Ripamonti 435, 20141 Milan, Italy; 3 Department of Biotechnology and Molecular Sciences, University of Varese, Via Dunant 3, 21100 Varese, Italy

**Keywords:** co-occurrence network, PubLiME, scale-free network, hierarchical modularity, date hub, party hub

## Abstract

Meta-analysis of high-throughput gene expression data is often used for the interpretation of proprietary gene expression data sets. We have recently shown that co-occurrence patterns of gene expression in published cancer-related gene expression signatures are reminiscent of several cancer signaling pathways. Indeed, significant co-occurrence of up to ten genes in published gene expression signatures can be exploited to build a co-occurrence network from the sets of co-occurring genes (“co-occurrence modules”). Such co-occurrence network is represented by an undirected graph, where single genes are assigned to vertices and edges indicate that two genes are significantly co-occurring. Thus, graph-cut methods can be used to identify groups of highly interconnected vertices (“network communities”) that correspond to sets of genes that are significantly co-regulated in human cancer. Here, we investigate the topological properties of co-occurrence networks derived from published gene expression signatures and show that co-occurrence networks are characterized by scale-free topology and hierarchical modularity. Furthermore, we report that genes with a “promiscuous” or a “faithful” co-occurrence pattern can be distinguished. This behavior is reminiscent of date and party hubs that have been identified in protein-protein interaction networks.

## Introduction

Current biological research is characterized by the application of high-throughput technologies which allow highly parallel studies of DNA, RNA, and protein functions to be carried out on an unprecedented scale. A major bottleneck in turning the large amounts of data accumulated into practically useful knowledge is the interpretation of the results. Comparative analyses of microarray-based gene expression studies can provide valuable insights, by helping in the interpretation of individual studies and pointing out unexpected parallels between studies (Larsson et al. 2006; Rhodes and Chinnaiyan, 2005). However, a number of technical hurdles, such as differences in the experimental procedures for sample collection, RNA extraction and labeling (Draghici et al. 2006) or differences in the microarray platforms used (Kuo et al. 2006) as well as the variety of statistical approaches employed during data analysis (Shi et al. 2005) make this type of analysis cumbersome.

We and others have recently proposed the use of gene list comparison approaches (Cahan et al. 2005; Finocchiaro et al. 2005; Newman and Weiner, 2005) to partially overcome these limitations, showing that meaningful conclusions can be drawn from published gene expression data in the absence of any numerical detail (Finocchiaro et al. 2007). Our approach is based on co-occurrence analysis. The underlying hypothesis assumes that genes regulated by similar pathways should co-occur more frequently that expected in published gene expression signatures. Thus, in order to systematically study co-occurrence patterns in gene expression signatures, we have generated a repository of published gene expression signatures, PubLiME (published lists of microarray experiments, available at http://bio.ifom-ieo-campus.it/publime) (Finocchiaro et al. 2007). We also proposed the Poisson-binomial distribution (which accounts for largely varying numbers of genes in reported gene lists) as an appropriate statistic to test the significance of co-occurrence of up to ten genes in published gene lists.

From the set of significantly co-occurring genes, a co-occurrence network is subsequently constructed as an undirected graph, with genes represented as vertices and edges indicating that two genes are significantly co-occurring (Finocchiaro et al. 2007). By this approach, we have shown that a co-occurrence network derived from cancer related gene expression signatures is characterized by the presence of highly interconnected communities, which can be identified using graph-cut approaches such as edge-betweenness clustering (Newman and Girvan, 2004).

Gene communities in the co-occurrence network are assumed to represent the consequence of coordinated differential regulation of the community genes in diverse conditions, which might be due to common regulatory inputs. Indeed, the promoters of community genes are characterized by over-represented transcription factor binding motifs, whose presence is compatible with biological intuition (Finocchiaro et al. 2007).

One of the most significant achievements obtained in recent years has been the realization that complex networks of biological entities are characterized by basic features that are also found in non-biological networks (Barabasi and Oltvai, 2004). Thus, physical insight derived from the study of non-biological complex systems may be used as a guide in the analysis of biological network function. Many naturally occurring networks possess the small-world property (Watts and Strogatz, 1998). Small-world networks are characterized by the contemporaneous presence of strong local clustering and short average path length between vertices. Such strong local clustering of the small-world model is consistent with the modularity observed in naturally occurring networks, where modules (i.e. communities of strongly interconnected vertices) are often observed (Ravasz and Barabasi, 2003). However, the small-world model cannot explain the vertex degree distribution of naturally occurring networks, which in the majority of cases follows a power law or an exponential law. On the other hand, the scale-free network model (Barabasi and Albert, 1999) explains the vertex degree distribution and still possesses the small-world property. However, local clustering in scale-free networks is much weaker than in naturally occurring networks. Therefore, the hierarchical network model has been proposed (Ravasz et al. 2002), which combines strong local clustering with small average path length (small-world property) and naturally observed vertex degree distribution (power law, i.e. scale-free property). Scale-free network topology has important implications for the robustness of complex systems (Albert et al. 2000). Since in scale-free networks most vertices have only a few edges, the accidental failure of vertices is likely to affect mainly the vertices themselves, without key roles for the function of the system. By contrast, the presence of hubs (vertices with many edges) makes scale-free networks particularly vulnerable because targeted removal of hub vertices quickly leads to disconnected subnetworks (Albert et al. 2000). These features are of obvious benefit in the search for new drug targets.

We were wondering whether co-occurrence networks derived from cancer related gene expression signatures share topological features common to other naturally occurring networks. If this would be the case, those features could be used in the identification of key regulators of the oncogenic process. We show here that co-occurrence networks are characterized by scale-free topology and hierarchical modularity. Furthermore, we identified two different co-occurrence patterns. Specifically, we found that some genes are differentially regulated in a wide variety of conditions and co-occur with many different genes. Paradoxically, this behavior leads to low vertex degrees in the co-occurrence network, since many co-occurrences never reached significance. Among those genes, we found well-known oncogenes playing a critical role in cancer, such as Cyclin D1 (CCND1) and FOS. On the other hand, we found genes that were less prone to differential regulation, but each time their expression level changed it did so in a coordinated fashion with a similar set of genes in different conditions. These genes represent the most connected hubs of the co-occurrence network. Examples of those genes are CDC2, CDKN3, and TK1. The significance of these findings in interpreting gene expression data and in identifying potential target genes for follow-up studies is discussed.

## Materials and Methods

### Generation of a repository of published cancer gene signatures

The generation of the PubLiME repository has been previously described (Finocchiaro et al. 2007). Briefly, 499 published cancer related gene expression microarray studies were scrutinized for: 1) aim of the study; 2) microarray platforms employed; 3) organism being investigated; and 4) feasibility of cross-platform annotation of published gene expression signatures. Among the 499 studies, 273 (233 human and 40 mouse) were selected for manual extraction of gene expression signatures from tables, figures, and supplementary material as lists of regulated genes. Cross-platform annotation was then performed as described (Finocchiaro et al. 2007). Data regarding publications and gene expression signatures were imported into a relational MySQL database that is accessible via a web-interface (http://bio.ifom-ieo-campus.it/publime/).

### Co-occurrence analysis of genes in gene expression signatures

Lists of regulated genes were represented in a bipartite graph format, where gene names and publication IDs represent the two vertex sets and an edge between them indicates differential regulation observed in a particular study. An edge-swapping procedure was applied in 1000 separate runs to determine the occurrence probability of a gene in a given publication. Given the occurrence probabilities for each gene in each publication, the probability of co-occurrences of arbitrary gene combinations (also called co-occurrence modules) in a publication could be calculated by multiplying the respective occurrence probabilities. The expected number of publications in which a gene combination is found follows a Poisson-binomial distribution (a binomial distribution with trial specific probabilities). Mean *μ* and variance *σ* of this distribution can be calculated as:

$$\mu =\sum_{i=1}^{N}p_{i}\;\text{and }\sigma =\sqrt[{2}]{\sum_{i=1}^{N}p_{i}-\sum_{i=1}^{N}p_{i}^{2}}$$

where *p_i_* designates the co-occurrence probability of a given gene combination in publication *i. N* is the total number of publications.

A Z-score transformation of the observed number *k* of co-occurrences of a given combination of genes can then be applied to assess the significance of co-occurrence.

$$Z=\frac{k-\mu }{\sigma }.$$

To limit noise effects, we required a co-occurrence module to be observed in at least five publications and the Z-score to be at least 5. A more detailed description of the analysis procedure can be found in Finocchiaro et al. 2007.

### Co-occurrence network construction

From the set of significant co-occurrence modules, a co-occurrence network was constructed in the following fashion: For each module, the gene names are represented by vertices and an edge is drawn between all pair-wise combinations of genes present in the module. This procedure is repeated for all significant co-occurrence modules.

### Regression analysis

Regression analysis was applied to estimate the scaling factors for the scale-free and exponential network models, as well as to investigate the relationship between the clustering coefficient C(k) and the vertex degree k.

In a scale-free network, the vertex degrees are distributed according to a power law:

$$P(k)∼k^{-\gamma }$$

where P(k) describes the probability of observing a vertex of degree k. γ is the scaling factor. After log transformation, this relationship becomes:

$$\text{ln}(P(k))∼-\gamma^{*}\text{ln}(k)$$

Thus, the relationship between ln(P(k)) and ln(k) is given by a line with slope −γ. To estimate γ, the observed data where plotted with ln(P(k)) on the y-axis and ln(k) on the x-axis and Mathematica software (“Fit” function) was used to find the equation of the line that best fits the data according to the least squares criterion. The slope of the regression line provides an estimate for the scaling factor γ. Regression analysis for the exponential network model was carried out similarly. However, since in an exponential network the vertex degrees follow an exponential law:

$$P(k)∼e^{-k\gamma }$$

which after log transformation becomes:

ln(P(k))∼-kγ

the data where plotted with ln(P(k)) on the y-axis and k (instead of ln(k)) on the x-axis before applying the Mathematica “Fit” function.

The clustering coefficient C(k) measures the fraction of observed edges divided by the number of theoretically possible edges linking direct vertex neighbors and thus offers a measure to detect modularity in networks. In hierarchical networks, the average clustering coefficient scales with C(k) ∼ k^−1^ and is independent of network size (Ravasz and Barabasi, 2003; Ravasz et al. 2002). Regression analysis was applied to verify this relationship in the PubLiME co-occurrence network, by plotting ln(C(k)) on the y-axis and ln(k) on the x-axis followed by applying the Mathematica “Fit” function to the data. The resulting regression line should have a slope close to −1 if the network is hierarchical.

### R-square value

The R-square value is calculated as:

$$R=1-\frac{\sum_{i=1}^{N}{\left(y_{i}-f_{i}\right)}^{2}}{\sum_{i=1}^{N}{\left(y_{i}-\mu \right)}^{2}}$$

*y_i_*—ith observed data value

*f_i_* – ith regression value

*μ* – data mean

*N* – number of data points

R-square assumes values between 0 and 1 and shows how much of the variability in the data is explained by the regression model. A value of 1 indicates a perfect fit.

### Functional gene category enrichment analysis

The DAVID database (Dennis et al. 2003) was used for functional category enrichment analysis, following the instructions given at http://niaid.abcc.ncifcrf.gov. The gene lists analyzed correspond to direct vertex neighbors of the genes studied. The multiple testing corrected P-values (Benjamini correction) are reported.

### Software

Custom Java based software was used for determining occurrence probabilities, co-occurrence probabilities and the identification of significant co-occurrence modules from PubLiME data. JUNG (http://jung.sourceforge.net/index.html) and Netsight (http://jung.sourceforge.net/netsight/) software were used for graph visualization. Mathematica software (Fit function) was used for linear regression analyses.

## Results

### Co-occurrence network vertex degrees are distributed non-randomly

**Figure 1:**
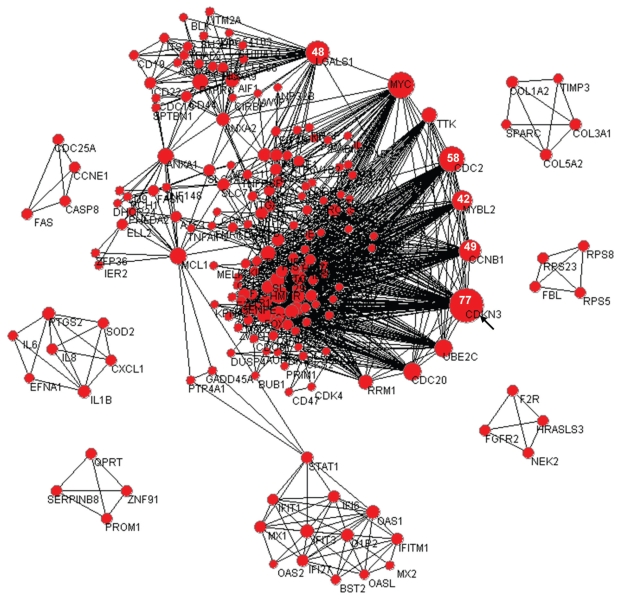
**The PubLiME co-occurrence network.** A representation of the PubLiME co-occurrence network is shown. The Z-score cutoff during co-occurrence analysis was set to 5 and co-occurrence modules of size 3 were required to be present in at least 5 publications. Larger vertex degrees are visualized by larger vertex diameter. The gene with the largest vertex degree (CDKN3) is indicated by an arrow and vertex degrees of the five most connected genes are shown in white letters.

Previously, we have reported co-occurrence analysis of published gene expression signatures collected in the PubLiME repository (Finocchiaro et al. 2005; Finocchiaro et al. 2007). From the set of significantly co-occurring genes, a co-occurrence network was constructed as described in Materials and Methods. A representation of the PubLiME co-occurrence network is shown in Figure 1. To investigate the topological properties of this network, we performed a vertex degree ranking analysis of the network as a first step (Fig. 1). The vertex diameter represents vertex degree (larger diameter indicates larger degree) and from this analysis the gene displaying the highest vertex degree is CDKN3 (77 edges), which is shown by an arrow. CDKN3 is a dual-specificity phosphatase that binds to cyclin-dependent kinases and inhibits cell cycle progression (Hannon et al. 1994). The next most connected genes are CDC2 (58 edges), CCNB1 (49 edges), LGALS1 (48 edges), and MYBL2 (42 edges). For these genes, the vertex degree is indicated in white letters in Figure 1. Without assuming a particular distribution of vertex degrees, a Z-score transformation of vertex degrees could be used to evaluate whether the vertex degrees of the above mentioned genes are compatible with a random distribution. Such Z-score transformation of vertex degrees was carried out by subtracting the mean vertex degree form the observed vertex degree, followed by dividing the result by the standard deviation of vertex degrees. The mean vertex degree of the network shown in Figure 1 was found to be 7.73, with a standard deviation of 9.42. Using these values, we obtained the Z-scores for the vertex degree of every gene. According to Tchebyshev’s theorem, the probability of observing these values by chance is at most the inverse of the square of Z-scores. These values are reported in Table 1.

This first analysis thus shows that the vertex degrees are not distributed in a random fashion.

### Analysis of co-occurrence network topology

**Figure 2:**
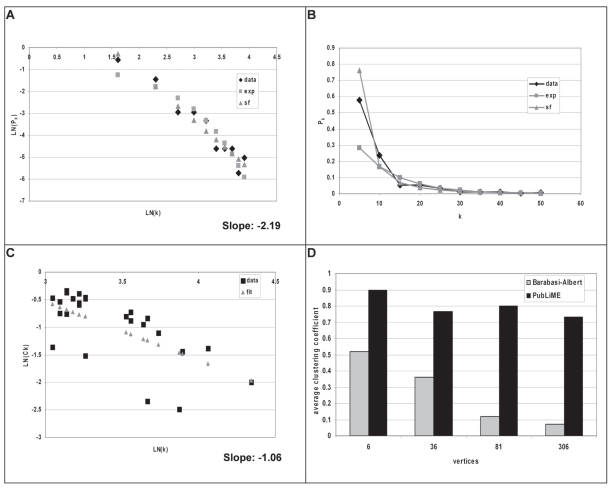
**PubLiME co-occurrence network topology.** **A**) The natural logarithm of the probability of observing a vertex with a given vertex degree category (<=5, 5 <= 10, 10 <= 15, 15 <= 20, 20 <= 25, 25 <= 30, 30 <= 35, 35 <= 40, 40 <= 45, 45 <= 50, <50) is plotted against the natural logarithm of vertex degrees (black diamonds). The slope of the line fitted to these data (the scaling parameter of the scale-free model (grey triangles)) by the least squares method is found to be −2.19. The exponential model (grey squares) has been obtained by fitting a line to the data in [k, ln(P(k)] linear-log space and is visualized here in [ln(k), lnN(P(k))] log-log space. exp-exponential model, sf-scale-free model. **B**) Observed vertex degree distribution (black diamonds) in [k, P(k)] linear-linear space along with the predicted vertex degree distributions according to the scale-free (grey triangles) and the exponential models (grey squares) are shown. **C**) The natural logarithm of the average clustering coefficient of vertices with the same degree is plotted against the natural logarithm of vertex degrees. Only vertices with degree above 20 were analyzed. The slope of the line fitted to these data using the least squares method (the scaling parameter) is found to be −1.06. **D**) The average clustering coefficient is shown for PubLiME co-occurrence networks derived for support 8, 7, 6, and 5. The support parameter indicates the minimal number of lists a module must be part of. The different support values cause the resulting networks to be of different sizes (number of vertices shown on the X-axis). Barabasi-Albert networks of equal size and degree distribution have been generated using the JUNG package random graph generator function for comparison purposes. The average clustering coefficient falls rapidly in Barabasi-Albert networks as network size grows. In PubLiME networks, the average clustering coefficient is stable.

Analysis of the PubLiME co-occurrence network’s topology produced the results shown in Figure 2. Figure 2A shows the distribution of the probability of observing a vertex with a given vertex degree P(k) as a function of the vertex degree k. The natural logarithm of both values is displayed and since P(k) <= 1, the values on the y-axis are negative. The data illustrate a linear relationship between the two variables (black squares) and show that high vertex degrees are less probable. Scale-free networks are characterized by the relationship P(k) ∼ k^−γ^ (γ = scaling coefficient), or ln(P(k)) ∼ −γ ln(k), i.e. an inverse linear relationship between ln(P(k)) and ln(k), as observed in the data. The slope of the line fitted to the data (grey triangles) using the least squares method (see Materials and Methods) evaluates to −2.19, which is typical for naturally occurring networks (Albert and Barabasi, 2002).

However, while many naturally occurring networks were found to be scale-free, some networks (e.g. transcription regulatory networks) turned out to be exponential (Barabasi and Oltvai, 2004). In exponential networks, the vertex degree distribution is described by the relationship P(k) ∼ e^−γk^. This function implies a linear relationship between ln(P(k)) and k, with slope −γ. In order to test whether the PubLiME co-occurrence network was better described by an exponential model, we fitted a line to the observed degree distribution in [k, ln(P(k))] space (see Materials and Methods) and visualized the result in [ln(k), ln(P(k)] space in Figure 2A (grey squares), in order to obtain a direct visual representation of the quality of fit for both the scale-free and exponential models. Visual inspection of the data showed that both the linear scale-free and the slightly curved exponential models fitted the data quite closely. In order to decide which model fits the data better, the results were then displayed in [k, P(k)] space (Fig. 2B) and the R-square value was calculated for both models. The R-square value indicates how much of the variation in the data is explained by the model. For the exponential model we obtained an R-square value of 0.68, while for the scale-free model the R-square value was 0.87. If R-square values are calculated in [ln(k), ln(P(k))] space, the corresponding values are 0.89 (exponential) and 0.96 (scale-free). Thus, the scale-free model explained the data much better than the exponential model and we concluded that the PubLiME co-occurrence network represented more likely a scale-free network than an exponential network.

To evaluate the modularity of the network, we also analyzed the scaling properties of the clustering coefficient C(k). In hierarchical networks, the average clustering coefficient scales with C(k) ∼ k^−1^ (Ravasz and Barabasi, 2003; Ravasz et al. 2002). Therefore, we tested whether the average clustering coefficient of the PubLiME co-occurrence network had this property. The results are shown in Figure 2C, which illustrates a linear relationship between ln(C(k)) and ln(k) (black squares). Although there are some outlier values, we observed that in the PubLiME co-occurrence network the clustering coefficient seemed to obey the C(k) ∼ k^−1^ rule. Regression analysis (grey triangles) was thus applied to estimate the scaling coefficient (see Materials and Methods) and we obtained a value of −1.06 (Fig. 2C) that was close to the theoretically expected scaling coefficient of −1.

As a further characteristic of hierarchical networks, it has been shown that the average clustering coefficient is usually much larger than in Barabasi-Albert networks having with similar degree distribution and is also largely independent of network size (Ravasz and Barabasi, 2003; Ravasz et al. 2002). In order to test whether these properties are present in the PubLiME co-occurrence network, we constructed co-occurrence networks from the PubLiME dataset, using different cutoff values for the support parameter S which requires a co-occurrence module to be observed in at least S publications. As a result, we obtained networks of different sizes and thus compared them to Barabasi-Albert networks with similar degree distribution and size generated by the JUNG random graph generator function. As can be seen in Figure 2D, the average clustering coefficient was largely independent of network size for the PubLiME co-occurrence networks, while it dropped rapidly in Barabasi-Albert networks. Thus, PubLiME networks apparently possess a scale-free topology with clear signs of hierarchical modularity.

### Hubs in the PubLiME co-occurrence network

We next asked whether differences in the co-occurrence patterns of genes can be identified. Previous analysis of the PubLiME dataset revealed that several genes display profound differences in their propensity of being detected as differentially regulated in a gene expression microarray experiment (Finocchiaro et al. 2007). Indeed, whereas the expression levels of some genes change in response to a wide variety of different biological conditions, most genes were found to display stable expression levels. For example, CCND1 was found to be differentially regulated in 15% of published studies, whereas two thirds of all human genes were never reported as differentially regulated. Thus, the question to be addressed is whether the genes that are most connected in the co-occurrence network are identical to the genes with the highest propensity to being differentially regulated in diverse conditions.

To investigate this question, the PubLiME dataset’s genes were sorted in descending order, according to both the total number of occurrences in the literature (Table 2) and the vertex degree in the co-occurrence network (Table 3). The top ten genes are shown in each case. Strikingly, the majority of genes that occur most frequently in the literature were not part of the co-occurrence network. This was because they did not co-occur consistently with other genes. Among these genes, some have known roles in oncogenesis, such as Cyclin D1 (CCND1), FOS and p21 (CDKN1A). Moreover, three of these genes co-occurred consistently with at least some genes (MYC, TNFAIP3, VEGF). However, with the exception of MYC, their vertex degrees were not exceptional. On the other hand, genes with highest vertex degrees were not ranked among the genes that occur most frequently in the literature (except for MYC, see Tables 2, 3). These results demonstrate that genes with the highest vertex degree in the co-occurrence network did not represent those which are most susceptible to underlie differential regulation. These data can be explained by assuming that the frequently regulated genes display a “promiscuous” behavior and co-occur in regulated gene lists with different genes in different conditions.

Assuming the correctness of the model described above, the vertex neighbors of frequently regulated genes should be characterized by heterogeneous functional gene annotations. We thus used the DAVID database (Dennis et al. 2003) (http://niaid.abcc.ncifcrf.gov/) to interrogate the set of vertex neighbors for enrichment of functional categories. The most significant category, along with the multiple testing corrected P-value as reported by DAVID is shown in Table 2. As reported, no coherent functional category could be identified for any of the frequently regulated genes. Surprisingly, however, nearly all genes with high vertex degree (Table 3) showed neighbors with consistent functional annotation. These results indicate that those genes not only co-occur with similar sets of genes in different conditions, but they also co-occur with genes playing similar roles in cellular physiology. In contrast to the promiscuous behavior of frequently regulated genes, they are thus “faithful” to a subset of genes which might be required for carrying out their function in a coordinated fashion.

In conclusion, two types of genes are likely distinguished in the PubLiME data set: 1) genes that respond to many different conditions by showing differential expression, but their expression is poorly correlated with the behavior of other genes, or 2) genes that are differentially expressed in fewer conditions, but their differential expression is often accompanied by differential expression of similar sets of genes. We can conclude that the PubLiME co-occurrence network is mainly dominated by faithful genes, whose function can generally be predicted from the function of their neighbors. Promiscuous genes, among which there are key regulators of the oncogenic process, are instead under-represented and their neighbors do not display functional consistency.

## Discussion

In this work, we investigated the topological properties of PubLiME co-occurrence networks. PubLiME is a database storing published gene expression signatures in a gene lists format (Finocchiaro et al. 2005; Finocchiaro et al. 2007). Other researchers have previously reported the development of similar resources (Cahan et al. 2005; Newman and Weiner, 2005). Gene expression studies are widely applied in order to shed light on several biological processes. However, standardized procedures on how to identify the biologically meaningful pieces of data in generally quite large datasets are still missing. A common procedure is to use gene category enrichment analysis on lists of differentially regulated genes to identify biological processes that are affected by a given biological condition. However, since this procedure relies on preassembled gene lists, new pathways cannot be identified. An additional problem is posed by the annotation quality of databases (Khatri et al. 2005). Furthermore, once a gene list has been found to be significantly associated with a given pathway, it is not clear which of the tens or hundreds of genes in the list are critically involved in regulating the pathway.

Within this frame, topological analysis of co-occurrence networks may offer an interesting alternative for several reasons. First, pathway target genes can be identified using graph-cut approaches, without relying on pre-assembled gene lists. Second, the hubs in co-occurrence networks suggest interesting genes for more detailed analysis.

We have shown that the PubLiME co-occurrence network displays a scale-free vertex degree distribution. While biological networks derived from high-throughput protein-protein interaction data, metabolism, or protein domains have been known for some time to possess scale-free topology (Barabasi and Oltvai, 2004; Titz et al. 2004), these studies have been carried out on networks making reference to structural properties of the biological entities. On the other hand, the PubLiME co-occurrence network does not rely on structure-driven interactions. It is based instead on co-occurrences of genes in published gene expression signatures manually extracted from a wide variety of studies using model cell lines or patient tissues.

Furthermore, our analysis of the clustering coefficient suggests that the co-occurrence network possesses hierarchical modularity, a conclusion that is compatible with previously reported gene communities identified in this network (Finocchiaro et al. 2007). It is worth noting that sampling quality can influence topology predictions quite significantly (Han et al. 2005). The PubLiME co-occurrence network has not been sampled, since the entire network has been analyzed for its topological properties. However, the PubLiME co-occurrence network is based on published gene expression signatures, and the collection of signatures accessible in PubLiME is necessarily incomplete, also because new signatures are being constantly produced. Therefore, future studies will be required to validate our conclusions.

It should also be recognized that the data collection in PubLiME is by design biased towards cancer-related gene expression signatures and may also be further biased in unknown ways, due to the experimental choices made by the researches whose signatures have been archived. Thus, the conclusions drawn about specific hub genes should be considered with caution and validated by future research. Nevertheless, we believe that the general conclusions about the hierarchical topology of co-occurrence networks of published gene expression signatures are not affected by these biases, since we have previously shown that gene communities correspond to different cancer signaling pathways and that the promoters of community genes are usually enriched for transcription factor binding motifs that are in line with biological intuition and experimentation (Finocchiaro et al. 2007). Moreover, we have shown that communities identified in humans correspond to communities identified in murine model systems (Finocchiaro et al. 2007). In other words, the hierarchical nature of the network seems to reflect biological reality. Thus, while the number and composition of communities will certainly be subject to changes as new signatures are being analyzed, the general topology is expected to remain hierarchical.

How could topological information of co-occurrence networks be used to identify interesting target genes for more detailed investigation? The scale-free nature of the co-occurrence network with a scaling parameter of 2.19 suggests that there are some hub genes having connections to a large part of the total of genes constituting the network. Indeed, CDKN3, a dual specificity phosphatase of cyclin dependent kinases that was discovered in the early 90’s (Hannon et al. 1994), is linked to 77 of the 306 genes (25%) in the network. These data suggest that CDKN3 plays a key role in regulating cell division. Interestingly, CDKN3 displayed more connections than CDC2 (58 edges), a *bona fide* key regulator of cell cycle progression. In general, hubs of the co-occurrence network represent genes with consistent co-occurrence behavior over a set of conditions. They co-occur with similar sets of genes and represent the core of subnetworks or modules, whose function can be predicted from the functional annotations of the genes constituting the community. As such, they represent excellent candidates for follow-up studies. However, detailed investigation of hub genes in the co-occurrence network has revealed that they differ in their propensity to co-occur with similar sets of genes. We noticed that the genes that were most frequently reported as differentially regulated in the literature were not among the genes that are most connected in the co-occurrence network. This observation suggests a “promiscuous” co-occurrence pattern for those genes. Interestingly, among them we find some known oncogenes such as CCND1 and FOS. It should be noted that most of these genes (with the exception of MYC) are not hubs in the co-occurrence network. They are referred to as hubs here simply because they are the most frequently occurring genes in the PubLiME dataset. In other words, they are occurrence hubs rather than co-occurrence hubs.

On the other hand, the genes with most edges in the co-occurrence network are not among the genes that are most often reported in lists of differentially regulated genes. However, when they are found differentially regulated, similar sets of genes are often found co-regulated. Thus, they are “faithful” to a subset of differentially regulated genes. Furthermore, the co-regulated genes are often characterized by similar functional annotations as the hub itself.

Taken together, these data suggest the presence of occurrence hubs and co-occurrence hubs in the PubLiME dataset, which often do not represent the same genes. Such a behavior is reminiscent of date and party hubs (Han et al. 2004). Party hubs tend to associate with similar sets of vertices in various conditions and are thought to represent structural organizers of semi-autonomous network modules. Date hubs, on the other hand, associate with different vertices in different conditions and might represent key regulators that orchestrate the activity of network modules according to the needs of a cell in specific circumstances. In a first approximation, promiscuous genes appear similar to date hubs while faithful genes behave more like party hubs. Given the observation that known oncogenes seem to behave as date hubs, it may be highly informative to study the behavior of other date hubs in cancer cells, in order to achieve interesting insight into the signaling pathways operating in cancer cells and the regulators influencing their function. The analysis of party hubs, on the other hand, may lead to the identification of novel drug targets whose inactivation might cause functional debilitation of downstream targets of deregulated signaling pathways, which in the co-occurrence network are forming communities of highly interconnected genes, or modules, with party hubs as central organizers.

## Figures and Tables

**Table 1:** Z-score and Tchebyshev limit of P-values for observing these vertex degrees by chance.

Gene	Z	P
CDKN3	7.351	0.019
CDC2	5.334	0.035
CCNB1	4.379	0.052
LGALS1	4.273	0.055
MYBL2	3.636	0.076

**Table 2:** Genes occurring most frequently in PubLiME.

Gene	Occurrences	Vertex degree	Clustering coefficient	DAVID category	Benjamini P-value
CCND1	30	0			
MYC	28	39	0.095816464	Cell cycle	0.42
TNFAIP3	26	14	0.142857143	Apoptosis	0.94
VEGF	25	9	0.333333333	Signal tranduction	1
CDKN1A	25	0			
FN1	25	0			
IL8	25	0			
CLU	24	0			
FOS	24	0			
IGFBP4	23	0			

**Table 3:** Genes with highest co-occurrence network vertex degree.

Gene	Occurrences	Vertex degree	Clustering coefficient	DAVID category	Benjamini P-value
CDKN3	19	77	0.136021873	Cell cycle	1.60E−18
CDC2	16	58	0.24984876	Cell cycle	7.10E−31
CCNB1	17	49	0.237244898	M-phase	3.60E−17
LGALS1	20	48	0.083333333	Immune response	9.60E−03
MYBL2	12	42	0.331010453	Cell cycle	6.10E−14
MYC	28	39	0.095816464	Cell cycle	0.42
TK1	13	39	0.431848853	Cell cycle	5.80E−17
TOP2A	22	38	0.385490754	Cell cycle	2.90E−15
CDC20	14	35	0.482352941	Cell cycle	1.20E−16
TTK	10	35	0.41512605	Cell cycle	5.00E−21
